# Prospective Evaluation of Clinical Outcomes Using a Multiplex Liquid Biopsy Targeting Diverse Resistance Mechanisms in Metastatic Prostate Cancer

**DOI:** 10.1200/JCO.21.00169

**Published:** 2021-07-01

**Authors:** Jamie M. Sperger, Hamid Emamekhoo, Rana R. McKay, Charlotte N. Stahlfeld, Anupama Singh, Xinyi E. Chen, Lucia Kwak, Cole S. Gilsdorf, Serena K. Wolfe, Xiao X. Wei, Rebecca Silver, Zhenwei Zhang, Michael J. Morris, Glenn Bubley, Felix Y. Feng, Howard I. Scher, Dana Rathkopf, Scott M. Dehm, Toni K. Choueiri, Susan Halabi, Andrew J. Armstrong, Alexander W. Wyatt, Mary-Ellen Taplin, Shuang G. Zhao, Joshua M. Lang

**Affiliations:** ^1^ Department of Medicine, Carbone Cancer Center, University of Wisconsin, Madison, WI; ^2^ Moores Cancer Center, University of California San Diego, La Jolla, CA; ^3^ Department of Urologic Sciences, Vancouver Prostate Centre, University of British Columbia, Vancouver, BC, Canada; ^4^ Dana-Farber Cancer Institute, Harvard Medical School, Boston, MA; ^5^ Memorial Sloan Kettering Cancer Center and Weill Cornell Medical College, New York, NY; ^6^ Beth Israel Deaconess Medical Center, Boston, MA; ^7^ Division of Hematology and Oncology, Department of Medicine, Helen Diller Family Comprehensive Cancer Center, University of California San Francisco, San Francisco, CA; ^8^ Department of Radiation Oncology, University of California San Francisco, San Francisco, CA; ^9^ Department of Urology, University of California San Francisco, San Francisco, CA; ^10^ Departments of Laboratory Medicine and Pathology and Urology, Masonic Cancer Center, University of Minnesota, Minneapolis, MN; ^11^ Duke Cancer Institute Center for Prostate and Urologic Cancers, Duke University, Durham, NC; ^12^ Department of Biostatistics and Bioinformatics, Duke University, Durham, NC; ^13^ Department of Human Oncology, University of Wisconsin, Madison, WI; ^14^ William S. Middleton Memorial Veterans Hospital, Madison, WI; ^15^ Department of Medicine, University of Wisconsin, Madison, WI

## Abstract

Nearly all men with prostate cancer treated with androgen receptor (AR) signaling inhibitors (ARSIs) develop resistance via diverse mechanisms including constitutive activation of the AR pathway, driven by AR genomic structural alterations, expression of AR splice variants (AR-Vs), or loss of AR dependence and lineage plasticity termed neuroendocrine prostate cancer. Understanding these de novo acquired ARSI resistance mechanisms is critical for optimizing therapy.

**MATERIALS AND METHODS:**

A novel liquid biopsy technology was used to collect mRNA from circulating tumor cells (CTCs) to measure expression of AR-Vs, AR targets, and neuroendocrine prostate cancer markers. An institutional review board–approved prospective cohort (N = 99) was used to identify patterns of gene expression. Two prospective multicenter phase II clinical trials of ARSIs for men with castration-resistant prostate cancer (ClinicalTrials.gov: NCT01942837 [enzalutamide, N = 21] and NCT02025010 [abiraterone, N = 27]) were used to further validate these findings.

**RESULTS:**

Hierarchical clustering of CTC transcripts identified two distinct clusters. Cluster 2 (C2) exhibited increased expression of AR-regulated genes and was associated with worse overall survival (median 8.6 *v* 22.4 months; *P* < .01; hazard ratio [HR] = 3.45 [1.9 to 6.14]). In multivariable analysis, C2 was prognostic independent of other clinicopathologic variables. AR-V status was not significant when accounting for C2. Upon further validation in pooled multicenter phase II trials, C2 was associated with worse overall survival (15.2 months *v* not reached; *P* < .01; HR = 8.43 [2.74 to 25.92]), prostate-specific antigen progression-free survival (3.6 *v* 12 months; *P* < .01; HR = 4.64 [1.53 to 14.11]), and radiographic progression-free survival (2.7 *v* 40.6 months; *P* < .01; HR = 4.64 [1.82 to 17.41]).

**CONCLUSION:**

We demonstrate that a transcriptional profile detectable in CTCs obtained from liquid biopsies can serve as an independent prognostic marker beyond AR-V7 in patients with metastatic prostate cancer and can be used to identify the emergence of multiple ARSI resistance mechanisms. This is currently being investigated in additional prospective trials.

## INTRODUCTION

Androgen Receptor (AR) signaling inhibitors (ARSIs), including abiraterone acetate (AA), enzalutamide, apalutamide, and darolutamide, have improved survival for men with metastatic castration-sensitive prostate cancer (CSPC) and castration-resistant prostate cancer (CRPC).^1-5^ However, approximately 5%-10% of patients will have primary resistance to ARSI treatment and a majority of initial responders will develop resistance within 1-3 years.^6-8^ The underlying drivers of treatment resistance can include activating AR genomic alterations (amplifications, mutations, and rearrangements), epigenetic alterations, and expression of truncated constitutively active AR splice variants (AR-V), among others.^7,9-11^ These alterations culminate in increased AR transcriptional activity and target gene expression despite androgen blockade. Conversely, some patients with CRPC develop AR-independent, neuroendocrine prostate cancer (NEPC) as an alternate escape pathway from androgen blockade.^12-15^ Comprehensive understanding of the molecular drivers of ARSI resistance and the ability to monitor this evolution over time is critical for early detection and appropriate treatment selection to improve outcomes for these patients.

**CONTEXT**

**Key Objective**
Multiple mechanisms of resistance result in disease progression in men with prostate cancer treated with androgen receptor (AR) targeted therapies (AR signaling inhibitor [ARSI]). This study aimed to develop a multiplex liquid biopsy that could detect resistance from AR-driven and AR-independent mechanisms to identify diverse drivers of treatment resistance.
**Knowledge Generated**
A pattern of gene expression was identified by hierarchical cluster analysis from RNA extracted from circulating tumor cells (CTCs) from patients with prostate cancer. This cluster was shown to be prognostic for decreased overall survival in the training cohort and decreased progression-free survival and overall survival in the validation cohort including patients from two clinical trials with ARSI.
**Relevance**
Detection of androgen receptor splice variant 7 in CTCs has emerged as a biomarker that is prognostic for treatment resistance to ARSI. Here, we demonstrate that expression of AR-regulated genes in CTCs is also prognostic for survival and radiographic progression that may have broader ability to identify treatment resistance than androgen receptor splice variant 7 alone.

Molecular profiling to detect these resistance mechanisms has historically required serial tumor biopsies, which carry a risk of procedural complication and high likelihood of not obtaining adequate tissue for analysis. The predominance of bone metastases in prostate cancer and the process of decalcification for pathologic assessment also render it challenging to assess RNA expression in metastatic bone biopsy samples. Liquid biopsies, including circulating tumor cells (CTCs) and circulating tumor DNA (ctDNA), have shown prognostic relevance in CRPC and the potential to be used as a surrogate biomarker of survival.^16-19^ *AR-V7* expression or protein in CTCs can be predictive of response to ARSIs such as enzalutamide and abiraterone acetate (AA).^20-26^ In the PROPHECY trial, which profiled 118 patients with CRPC, 0% and 11% of patients who had detectable CTC *AR-V7* by the Epic Sciences nuclear AR-V7 protein assay or the Johns Hopkins *AR-V7* mRNA assay, respectively, had confirmed prostate-specific antigen (PSA) responses. The AR-V7–positive patients also had significantly worse progression-free and overall survival (OS) as compared with patients who lacked detectable AR-V7. The prevalence of AR-V7 is low in the first-line setting and many AR-V7–negative patients do not respond to second-line ARSIs, suggesting other mechanisms of resistance.^26-28^ NEPC is characterized by the loss of *AR* transcriptional activity in tandem with upregulation of neuroendocrine markers and is observed in 5%-17% of men with CRPC.^14,15,29^ NEPC has been previously identified using ctDNA methylation profiling and morphologic characterization of CTCs.^30,31^ To our knowledge, there is no assay capable of assessing AR-Vs, AR activity, and NEPC on a single platform, which is required to understand the timing and emergence of these diverse resistance mechanisms that occur and interact.

Herein, we present a CTC liquid biopsy transcriptional assay capable of simultaneously measuring these three resistance mechanisms. This multiplex panel includes AR splice variants (*AR-V7* and *AR-V9*), a panel of AR-regulated genes (*TMPRSS2*, *KLK2*, *KLK3*, and *FOLH1*) that may indicate persistent or overactivation of AR transcriptional activity, and NEPC-associated genes (*SYP* and *CHGA*). We applied this assay to a large multi-institutional cohort of patients with metastatic prostate cancer where we observed associations with clinical outcomes and described longitudinal changes compared with changes in ctDNA. These findings were subsequently independently validated in two multicenter phase II trials.

## MATERIALS AND METHODS

### Multi-Institutional Prospective Cohort

Between August 2014 and January 2020, blood samples were collected from 99 patients with prostate cancer treated at the University of Wisconsin Carbone Cancer Center and Dana Farber Cancer Institute (DFCI). Patients were required to have a histologically confirmed metastatic prostate cancer diagnosis. All patients gave informed consent following an institutional review board–approved protocol to participate in prospective blood sample collection and analysis. Study data were managed using approved REDCap electronic database hosted at the University of Wisconsin (UW)–Madison, School of Medicine and Public Health.^32^ This cohort was used as the training set for the C2 classifier.

### Phase II Clinical Trials

The ENZA-CRPC trial (ClinicalTrials.gov: NCT01942837) was a phase II trial of enzalutamide in metastatic CRPC (mCRPC), which accrued 66 patients from 2013 to 2017. The AA-CRPC trial (ClinicalTrials.gov: NCT02025010) was a phase II trial of AA without glucocorticoid therapy for men with mCRPC,^33^ which accrued 58 patients between 2014 and 2016. Liquid biopsies were prospectively collected before treatment and evaluable on 21 patients from ENZA-CRPC and 27 patients from AA-CRPC, for a total of 48 patients from prospective phase II trials treated with ARSIs. These trials served as independent validation for the C2 classifier.

### CTC Isolation, RNA Extraction, and Quantitative Reverse Transcriptase-Polymerase Chain Reaction

Fifteen milliliters of blood was collected from each patient in EDTA vacutainer tubes (BD Biosciences, Franklin Lakes, NJ). Samples were shipped in temperature-controlled packing and processed within 24 hours. The VERSA platform, as described previously,^34^ was used for CTC capture and extraction of mRNA.^34,35^ Quantitative reverse transcriptase-polymerase chain reaction was used to assess gene expression. Details are available in the Data Supplement (online only). Expression values of CTCs isolated from 15 mL of blood were calculated as 38 − Ct values and were reported as raw (unnormalized) data.

### Circulating Tumor DNA

Plasma was collected from whole blood collected in EDTA tubes within 4 hours of draw and before isolation of CTCs. Plasma (6 mL) was removed after centrifugation at 290 × *g* for 10 minutes, spun at 2,730 × *g* for 10 minutes, and stored at –80°C. Cell-free DNA was isolated using the Qiagen Circulating Nucleic Acids kit following manufacturer's instructions. Cell-free DNA sequencing was performed as previously described.^36^

### Statistical Analysis

Hierarchical clustering of the gene expression data from the UW-DFCI samples (training set) was performed using the R hclust function with default parameters to identify distinct clusters. To create a single-sample approach to independently classify samples in the phase II trials, a nearest shrunken centroids classifier^37^ was trained using the same genes on the baseline CTC samples using the R pamr package with default parameters. This approach computes a centroid for each cluster, applies a shrinkage step to reduce noise, and classifies new samples on the basis of the nearest centroid. This was then locked and used to classify the ENZA-CRPC and AA-CRPC samples (independent validation set). For comparisons between two clusters, Fisher's exact test and Mann-Whitney U test were used for categorical and continuous variables, respectively. OS was the primary clinical end point and was defined as date of death or last contact relative to treatment start. PSA progression-free survival (PSA PFS) was defined as date of PSA progression according to the Prostate Cancer Working Group 2 criteria or death because of any cause or censored at date of last disease assessment relative to treatment start. Radiographic PFS (rPFS) was defined as radiographic progression according to RECIST 1.1 criteria for soft tissue and lymph node disease and Prostate Cancer Working Group 2 for bone disease or death because of any cause or censored at date of last disease assessment relative to treatment start. The Kaplan-Meier method was used to estimate the survival distributions by clusters and AR-V status, and log-rank test was used to compare two groups. Univariate and multivariate Cox proportional hazards models were fitted to quantify the association of molecular and clinical variables with time-to-event end points.

## RESULTS

Blood samples from 99 unique patients with metastatic prostate cancer were included in the initial cohort. Patient characteristics and treatment history at the time of blood sample collection are detailed in Table 1. The samples were acquired from a biospecimen protocol that collected blood from any patient with metastatic prostate cancer for correlation with clinical outcomes. As such, the patient cohort consisted of patients with CSPC (28%), CRPC (67%), and NEPC (6%) at the time of blood draw. These patients received various treatments, including androgen deprivation therapy, ARSIs, taxane chemotherapy, platinum doublet therapies, and clinical trials. Forty-eight percent of the patients had current or prior treatment with ARSIs, whereas 20% had current or prior treatment with taxane chemotherapy.

**TABLE 1.**
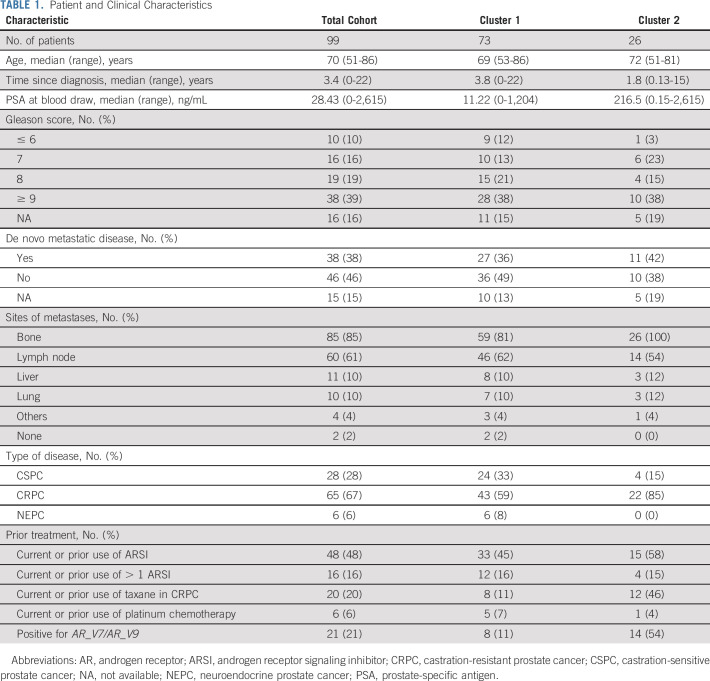
Patient and Clinical Characteristics

### Transcriptional Analysis of CTCs

**FIG 1.**
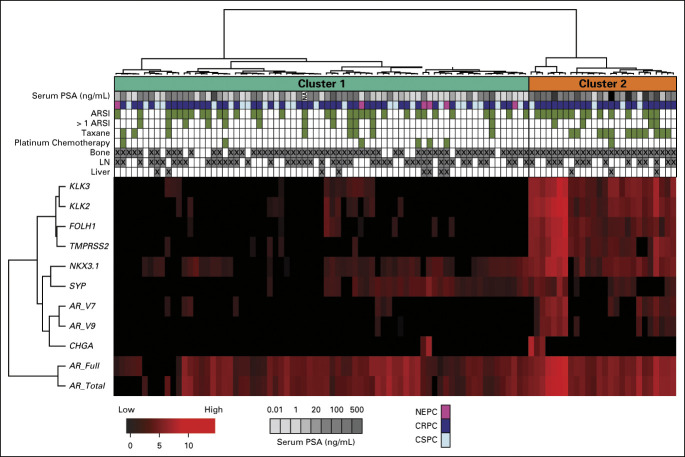
Two distinct molecular clusters in circulating tumor cells from patients with metastatic prostate cancer. Hierarchical clustering of gene expression data of 11 genes from 99 patients with prostate cancer identified two distinct clusters (cluster 1: C1 and cluster 2: C2). Serum PSA (ng/mL), prostate cancer type, treatment with ARSI or chemotherapy in CRPC setting, sites of metastases at time of blood draw, and serum PSA at time of blood draw are indicated. X indicates presence of metastases at indicted site. AR, androgen receptor; ARSI, androgen receptor signaling inhibitor; AR-V, androgen receptor splice variant; CRPC, castration-resistant prostate cancer; CSPC, castration-sensitive prostate cancer; LN, lymph node; NEPC, neuroendocrine prostate cancer; PSA, prostate-specific antigen.

Quantitative multiplex reverse transcriptase-polymerase chain reaction was used to measure the expression of *AR*, AR-Vs, AR-regulated genes, and genes upregulated in NEPC using RNA isolated from CTCs captured with an anti-EpCAM antibody. Hierarchical clustering of the transcript expression from these samples identified two distinct clusters of patients (Fig 1). Cluster 1 (C1) was characterized by low to absence of detection of AR-regulated genes. C1 included all patients with a histologic diagnosis of NEPC. By contrast, cluster 2 (C2) was enriched for patients who had high expression of transcriptional targets of AR, indicating increased AR transcriptional activity. Consistent with increased AR activity, patients classified as C2 had higher levels of serum PSA (median 216 ng/mL) relative to the patients in cluster 1 (median 11.22 ng/mL, *P* < .0001) (Data Supplement, Table 1). Patient and disease characteristics for each cluster are detailed in Table 1. Patient age, time since diagnosis, and percent of patients diagnosed with metastatic disease at initial diagnosis were all similar in C1 and C2. Patients in C2 were significantly more likely to have CRPC versus CSPC and bone metastases and had treatment with taxane chemotherapy.

### UW-DFCI Clinical Outcomes

**FIG 2.**
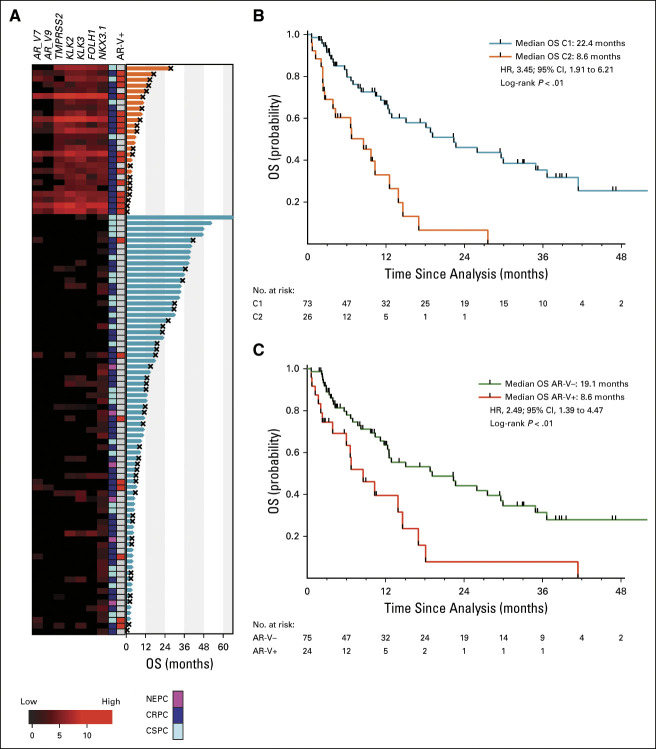
C2 is prognostic for OS independent of AR-V status. (A) Swimmer plot ordered by OS for C2 (orange, top) and C1 (cyan, bottom). Gene expression for AR-V and AR pathway genes is indicated. X indicates the patient death. (B) Kaplan-Meier plot of OS by C2 versus C1. (C) Kaplan-Meier plot of OS by AR-V status. AR, androgen receptor; AR-V, androgen receptor splice variant; C1, cluster 1; C2, cluster 2; CRPC, castration-resistant prostate cancer; CSPC, castration-sensitive prostate cancer; HR, hazard ratio; NEPC, neuroendocrine prostate cancer; OS, overall survival.

**FIG 3.**
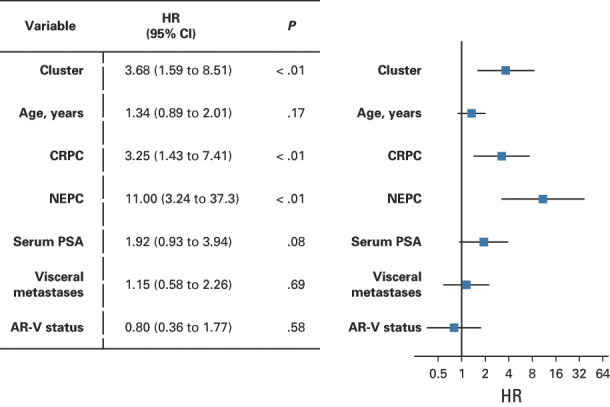
Multivariate survival analysis indicates that the association of cluster and risk of OS remained after adjusting covariates. Association of AR-V status and OS was no longer significant. AR-V, androgen receptor splice variant; CRPC, castration-resistant prostate cancer; HR, hazard ratio; NEPC, neuroendocrine prostate cancer; OS, overall survival; PSA, prostate-specific antigen.

We next examined the clinical end point of OS. At time of analysis, 55 patients (56%) were deceased and the median follow-up time was 9.4 months in this cohort. We observed significantly shorter OS for patients in C2 versus C1 (Figs 2A and 2B). The median OS was 8.6 months in C2 (95% CI, 2.6 to 12.6) (n = 26) versus 22.4 (95% CI, 12.5 to 34.9) months in C1 (n = 73) (*P* < .01, hazard ratio [HR] = 3.45 [1.91 to 6.21]). As has been reported,^20,22,26^ AR-V+ patients (*AR-V7* and *AR-V9*) had worse OS (Fig 2C). The median OS for AR-V+ patients was 8.6 (95% CI, 3.9 to 14.6) months (n = 24) versus 19.1 (95% CI, 12.3 to 30) months (n = 75) for AR-V– (*P* < .01, HR = 2.49 [1.39 to 4.47]). On multivariable analysis (MVA), C2 was independently associated with shorter OS after adjusting for patient age, CRPC versus CSPC, NEPC versus CSPC, PSA, visceral metastasis, and AR-V status. CRPC and NEPC were the only other variables also independently associated with OS on MVA (Fig 3 and Data Supplement). Consistent with the MVA, OS remained shorter for patients in C2 when patients with CRPC (Data Supplement) and CSPC (Data Supplement) were analyzed independently. Unexpectedly, AR-V status was not statistically significant in the MVA. AR-V+ patients were significantly enriched in C2 versus C1 (Fig 2A, 54% *v* 10%, *P* < .0001), suggesting that AR-V status may be prognostic because of its association with C2, rather than being independently prognostic. When we examined only C2 patients, no significant differences in OS were detected on the basis of AR-V status (Data Supplement). However, when we examined only AR-V+ patients, significantly shorter OS was observed for C2 patients compared with C1 patients (Data Supplement). This result is consistent with the MVA and suggests that these transcriptional clusters are inclusive of AR-Vs, and the prognostic importance of AR-Vs alone is eliminated when taking into account CTC gene expression clusters.

### Independent Validation in Prospective Trials With AA or Enzalutamide

**FIG 4.**
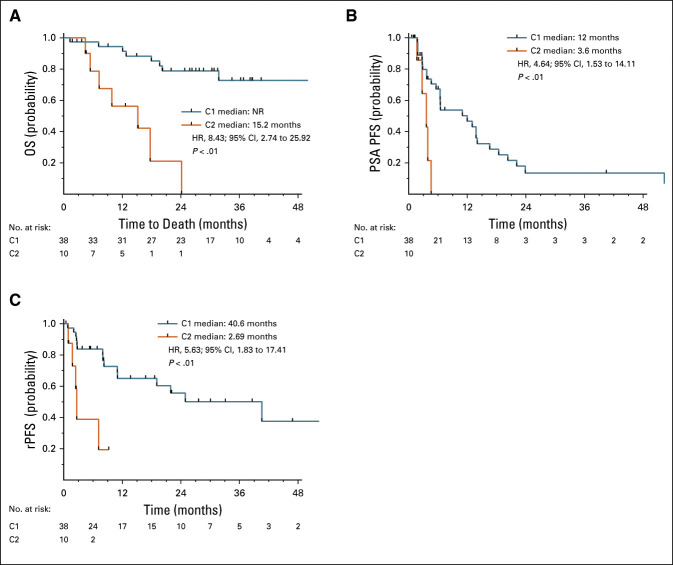
C2 has worse PSA, rPFS, and OS in two multicenter phase II trials of androgen receptor signaling inhibitors. Kaplan-Meier plot of (A) OS, (B) PSA PFS, and (C) rPFS for patients in C2 compared with C1 in patient treated with enzalutamide or abiraterone acetate. C1, cluster 1; C2, cluster 2; HR, hazard ratio; NR, not reached; OS, overall survival; PFS, progression-free survival; PSA, prostate-specific antigen; rPFS, radiographic progression-free survival.

We next sought to validate our findings in two prospective phase II trials of patients with mCRPC (excluding NEPC) treated with ARSIs, enzalutamide (ENZA-CRPC), or AA-CRPC. At time of analysis, 15 patients (31%) were deceased and the median follow-up time was 28 months in this cohort. We applied the classifier trained in UW-DFCI to these cohorts to independently identify and clinically validate C2-like patterns in CTCs. In the combined trials of ENZA-CRPC and AA-CRPC (N = 48), we identified 10 patients (21%) with a C2 expression pattern. This C2 cluster was associated with worse OS (median 15.2 [95% CI, 4.4 to not reached (NR)] months *v* NR; *P* < .01; HR = 8.43 [2.74 to 25.92]) (Fig 4A) in the pooled validation cohort. These independent results are consistent with the findings in our multi-institutional UW-DFCI cohort. Similarly, C2 is independently prognostic for OS relative to serum PSA, the presence of visceral metastases, age, and AR-V status using pairwise MVA in the phase II trials (Data Supplement). Importantly, we also observed that patients in C2 had significantly shorter PSA PFS (3.6 [95% CI, 1.7 to NR] *v* 12 [95% CI, 5.6 to 14] months; *P* < .01; HR = 4.64 [1.53 to 14.11]) (Fig 4B) and rPFS (2.7 [95% CI, 1 to NR] *v* 40.6 [95% CI, 11 to NR] months; *P* < .01; HR = 5.63 [1.82 to 17.41]) (Fig 4C).

### CTC and ctDNA Changes Precede Clinical Progression

**FIG 5.**
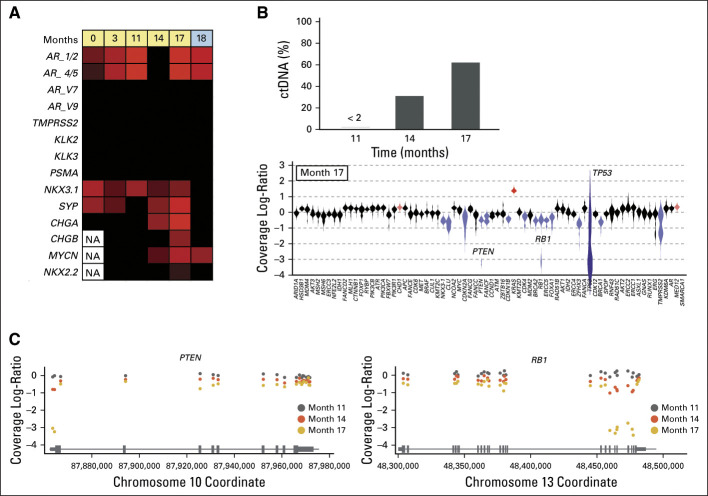
Treatment-emergent NEPC can be detected before the onset of clinical progression. (A) Heatmap of gene expression data demonstrates the acquisition of genes commonly upregulated in NEPC as a patient is treated with androgen deprivation therapy (yellow) and carboplatin and etoposide (blue). (B) Top panel shows ctDNA fractions (as a proportion of total cell-free DNA) at three sampled timepoints. Bottom panel shows a violin plot of the copy number profile at month 17. Blue indicates genes that pass the thresholds for identifying a copy deletion, and red indicates copy gain. Note that the violin for TP53 is distorted because of very low coverage (because of a deep deletion). For *PTEN* and *RB1*, the violins have long tails, suggesting that there may be intragenic differences in copy profiles. (C) Copy number profiles showing *RB1* and *PTEN* exon-level data. All three patient timepoints are shown. Differences between the timepoint are a function of ctDNA fraction. Note that the high ctDNA fraction in the month 17 timepoint enables identification of an exon 1 deep deletion in *PTEN* and a 3′ deep deletion within *RB1*. AR, androgen receptor; AR-V, androgen receptor splice variant; ctDNA, circulating tumor DNA; NA, not available; NEPC, neuroendocrine prostate cancer.

An advantage of liquid biopsy technology is the ability to collect serial samples. We obtained longitudinal CTC and ctDNA data from a patient with CSPC. The patient initially presented with widespread bone metastases and a single liver lesion with a serum PSA of 175 ng/mL. A bone biopsy showed a poorly differentiated adenocarcinoma that was positive for PSA and prostatic acid phosphatase. The patient was treated with combined androgen blockade and docetaxel. Serum PSA decreased to 0.33 ng/mL 1 year after the initial presentation, and complete resolution of the liver lesion was noted. At the 17-month timepoint, the patient developed pancytopenia and elevated liver enzymes, whereas restaging scans identified extensive new bone, liver, and lung metastases. A liver biopsy was performed that showed emergence of NEPC with expression of synaptophysin and chromogranin A, and Ki67 was 80% positive. When we examined gene expression in the patient's longitudinal CTC samples, we observed acquired expression of *SYP*, *MYCN*, and *CHGA* 3 months before clinical symptoms (Fig 5A). These molecular changes in CTCs mirrored changes in ctDNA content, which increased from months 14 to 17. NEPC is enriched for inactivating alterations in *TP53*, *RB1*, and *PTEN*.^14,15^ We observed focal copy number loss in liquid biopsies from months 11 to 17 for all three and concordant expression of *NKX2.2* and *CHGB* (Figs 5B and 5C). The timing of the CTC and ctDNA changes was concordant, and both preceded clinical progression at month 17. These data demonstrate that integration of ctDNA and CTC molecular profiling is feasible and can promote early identification of NEPC emergence to improve patient stratification for clinical trials and novel therapeutic strategies.

## DISCUSSION

Liquid biopsies using CTCs or ctDNA represent an attractive alternative to tissue-based molecular profiling. Herein, to our knowledge, we present the first study of a multiplex gene expression biomarker panel in 147 patients with metastatic prostate cancer designed to detect three major mechanisms of resistance to ARSIs in prostate cancer: AR-Vs, AR pathway activation, and NEPC. Although landmark studies of liquid biomarkers for AR-Vs or NEPC individually have been published previously,^20,23,25,26,30^ to our knowledge, this is the first report of an assay capable of measuring all these components of resistance simultaneously. When we applied this CTC assay to a large cohort of patients with metastatic prostate cancer, we identified a distinct cluster C2 characterized by high expression of AR target genes, which was prognostic for OS independent of other variables including AR-V status, CRPC, and NEPC. We then independently validated the prognostic significance of C2 in a combination of two phase II CRPC ARSI trials.

Many diverse mechanisms of resistance to androgen blockade culminate in increased expression of AR target gene expression. These include mutations, amplifications, and rearrangements of the AR gene;^38-44^ amplification of an enhancer element upstream of the AR gene;^44,45^ expression of AR-Vs such as *AR-V7* or *AR-V9*;^10,20,46,47^ epigenetic modifications (eg, methylation and BET proteins),^48-50^ or even bypassing AR completely via glucocorticoid receptor.^51^ C2 appears to be primarily driven by increased expression of AR-regulated genes, whereas total AR levels do not differ dramatically. Interestingly, our work suggests that prior interest in AR-Vs may be incomplete. AR-V positivity was correlated with C2, but after adjusting for C2 membership, AR-V status was no longer prognostic. This may be because multiple mechanisms can result in ARSI resistance and manifest in increased AR signaling activity, including not only AR-Vs but also from a myriad of other etiologies. We also independently validated that C2 was associated with worse prognosis in two phase II ARSI trials. Strikingly, C2 was also associated with worse PSA and rPFS with ARSIs, suggesting that it could be used as a biomarker to identify patients who may benefit from additional or alternative therapies up-front and monitor for the early emergence of molecular resistance. These findings warrant further exploration in larger prospective trials.

Previous studies of CTCs in CRPC identified other prognostic signatures that associate with clinical outcomes. CTC enumeration has been shown to be prognostic for OS in prostate cancer using Cell Search platform.^16-18^ The PROPHECY trial demonstrated that AR-V7 expression, either by gene expression and protein analysis, is prognostic for PFS and OS with ARSI treatment independent of enumeration.^26^ It is unknown if the number of CTCs present in each sample may affect the detection or level of gene expression for the genes in this panel. Further analysis is necessary for the panel in this report to evaluate the relative contribution of CTC number to this assay. In addition, broader evaluation of non–AR-driven resistance mechanisms, such as the recently described Double Negative Prostate Cancer classifier, may identify a distinct population of patients with different outcomes following treatment with ARSIs. Expansion of this gene expression panel to incorporate newly identified disease subtypes and mechanisms of resistance may further enhance the utility of liquid biopsies.

Molecular classifiers such as the approach described in this study also represent an attractive way to follow prostate cancer disease status in addition to PSA and imaging. These could be easily integrated into existing clinical workflows, such that, in addition to standard periodic laboratory studies, blood samples could be sent for real-time liquid biomarker studies. We demonstrate the feasibility and concordance of longitudinal CTC and ctDNA profiling simultaneously. Identification of molecular mechanisms of ARSI resistance could trigger a change in therapy or enrollment into clinical trials. Early detection maximizes eligibility for other treatments, and although tumor burden is still low (eg, at month 14, before widespread clinical progression at month 17 in the patient described), the patient's performance status is still optimal, before symptomatic disease progression.

In conclusion, to our knowledge, we demonstrate the first multiplex gene expression liquid biopsy assay that can assess multiple potential ARSI resistance mechanisms simultaneously in metastatic prostate cancer. Early identification of these molecular changes could help guide treatment decisions. The heterogeneous nature of this cohort, which included different disease states and therapies, suggests that these findings may be generalizable to the broader patient population. The validation of our prognostic clustering in two phase II trials with ARSIs suggests clinical utility; however, larger clinical trials are necessary. We are prospectively testing these findings further in multiple ongoing clinical trials for patients with CSPC and CRPC (NCT02445976, NCT01942837, NCT03725761, NCT02025010, and NCT04126070).

## Data Availability

Individual participant data that are included in the results reported in the manuscript will be available after deidentification beginning 6 months and ending 36 months following publication. Researchers must provide a methodologically sound proposal, and requestors may need to sign a data access agreement. Proposals should be directed to the corresponding author.
